# Genotyping on ALDH2: Comparison of Four Different Technologies

**DOI:** 10.1371/journal.pone.0122745

**Published:** 2015-03-24

**Authors:** Lina Zhang, Jinzhao Zhao, Guanglin Cui, Hong Wang, Dao Wen Wang

**Affiliations:** Departments of Internal Medicine, Tongji Hospital, Tongji Medical College, Huazhong University of Science and Technology, Wuhan, People’s Rep. of China; Chinese Academy of Medical Sciences, CHINA

## Abstract

**Objectives:**

This study aimed to compare the accuracy and performance of four genotyping methods for detecting single nucleotide polymorphisms (SNPs) in aldehyde dehydrogenase-2 (ALDH2), which is the principal enzyme involved in alcohol metabolism.

**Design and Methods:**

We genotyped rs671 of ALDH2 in 96 coronary heart disease (CHD) patients with four methods including high resolution melting analysis (HRM), TaqMan allelic discrimination assay (TaqMan), allele-specific PCR (AS-PCR) and pyrosequencing. Meanwhile, we compared the accuracy and performance of these methods.

**Results:**

All selected patients were successfully genotyped with referred methods. The results of these four assays showed 100% concordant results and had 100% accuracy as verified by Sanger sequencing.

**Conclusions:**

All of the referred methods can be used for genotyping ALDH2 rs671 with the same accuracy compared to Sanger sequencing. In small size of clinical samples, HRM and AS-PCR outperform over others due to their lower cost and less hands-on operation, which are suitable for clinical application.

## Introduction

Aldehyde dehydrogenase-2 (ALDH2) is the second enzyme of the major oxidative pathway of alcohol metabolism. It plays a pivotal role in alcohol metabolism in human beings [1, 2]. Emerging evidence has shown that a common single nucleotide polymorphism (SNP) rs671 G>A (Glu504Lys) in gene ALDH2 influences enzymatic activity of ALDH2 [3, 4]. It has been reported that rs671 is associated with susceptibility to coronary heart disease (CHD) [5]. During the past years, a variety of genotyping techniques have been developed for identifying rs671, such as restriction fragment length polymorphism, single-stranded conformational polymorphism or denaturing high performance liquid chromatography [6–8]. Although the results of these methods are reliable, they tend to be time consuming, expensive and/or labor intensive in practice, making them unsuitable for routine clinical application. Therefore, it is necessary to develop both time and cost-effective as well as robust techniques for rs671 genotyping. In this study, we applied four different methodological approaches, including high resolution melting analysis (HRM), TaqMan allelic discrimination assay (TaqMan), allele-specific PCR (AS-PCR) and pyrosequencing to genotype rs671 in patients with CHD. We aimed to compare the accuracy, robustness and cost of these four methods with a focus on their clinical application.

## Materials and Methods

### 1 Study subjects and DNA samples

In our study, 96 CHD patients (48 males and 48 females) confirmed by coronary angiography were recruited from Tongji Hospital in Wuhan (Hubei, China) in 2012. CHD was defined as luminal stenosis ≥50% in at least one major coronary artery branch. All patients provided written informed consent and the study was approved by the institutional review board of Tongji hospital. The work was carried out in accordance with the Code of Ethics of the World Medical Association (Declaration of Helsinki) for experiments involving humans.

Blood samples were drawn from all participants and genomic DNA was extracted from the leukocytes using a DNA isolation kit in accordance with the protocol (TIANGEN biotech, Beijing) and quantified by a NanoDrop 2000 Spectrophotometer (NanoDrop Technologies, Wilmington, DE). These samples had a final concentration ranging from 10 to 30 ng /μl. All assays were performed in duplicate.

### 2 HRM

Genotyping of rs671 by HRM was performed in 384-well plates on a 384-well 7900HT Fast Real-Time PCR System (7900 HT, Applied Biosystems, Foster City, CA) as previously described [9]. Briefly, two primers (as shown in Table 1) were designed to amplify a 78 bp fragment cover target locus rs671 and SYTO 9 (Invitrogen, Eugene, Oregon, USA), a saturating dye was added to the standard PCR reaction mixture before amplification. The PCR reaction mixture contained 5 μl 2x GC Buffer, 6.25 μmol of each dNTP, 0.25 U Taq DNA Polymerase (TaKaRa Biotechnology, Tokyo, Japan), 1 μl genomic DNA, 100 pmol SYTO 9 and 5 pmol of each primer in a reaction volume of 10 μl. PCR conditions were as follows: initial denaturation at 95°C for 10 min, followed by 40 cycles of denaturation at 95°C for 15 sec, annealing/extending at 60°C for 1 min. After PCR, the samples were melted at a ramp rate of 1% from 60°C to 95°C. The fluorescence signals were measured during this process and the melting curve was automatically converted to melting peaks. SDS Software v2.3 and High Resolution Melt v2.0 (Applied Biosystems, Foster City, CA) were used for data analysis.

**Table 1:** Primers and probes used for ALDH2 genotyping.

methods		sequence
HRM	forward primer	5' GGGGAGTGGCCGGGAG 3'
	reverse primer	5' CCAGCAGGTCCCACACTCAC 3'
TaqMan	forward primer	5' GGCTACAAGATGTCGGGGAG 3'
	reverse primer	5' AGACCCTCAAGCCCCAACA 3'
	probe1	FAM-CATACACTGAAGTGAAAA—MGB
	probe2	HEX-CATACACTAAAGTGAAAAC-MGB
AS-PCR	common primer	5' CATAACCCCCAAGAGTGATTTC 3'
	G allele-special primer	5' CCCACACTCACAGTTTTCACATC 3'
	A allele-special primer	5' CCCACACTCACAGTTTTCACATT 3'
Pyrosequencing	forward primer	5' Biotin-GGGAGTTGGGCGAGTACG 3'
	reverse primer	5' CCCCAACAGACCCCAATC 3'
	sequencing primer	5' CCACACTCACAGTTTTCACT 3'
Sanger sequencing	forward primer	5' CATAACCCCCAAGAGTGATTTC 3'
	reverse primer	5' AGAGCAGAGGCTGGGTCTTTAC 3'

### 3 TaqMan

TaqMan [10] was also performed using a 7900 HT with primers and probes synthesized by ABI (Applied Biosystems, Foster City, CA). The primers and probes are shown in Table 1. Specific probes for different alleles were labeled at their 5′-end with the fluorescence reporter dyes FAM and HEX, respectively. Reactions were performed in 5 μl comprising 2.5 μl TaqMan universal PCR master mix (Applied Biosystems, Foster City, CA), 5 pmol TaqMan primers, 1 pmol probes and 1 μl DNA. PCR cycling conditions included 50°C for 2 min, 95°C for 10 min followed by 45 cycles of 95°C for 15 s, 60°C for 1 min. The fluorescence signals were collected during amplification. Data was analyzed using SDS Software v2.3.

### 4 AS-PCR

For analysis of rs671, a common forward primer and two allele-specific reverse primers were designed, with the 3′ base of each reverse primer corresponding to either allele G or A of rs671, respectively. Depending on the sample genotype, either one or the other or both allele-specific primer(s) can amplify the target sequence. To enhance the specificity of the assay, we employed a mismatch at the third 3’ base of both reverse primers [11] (as shown in Table 1). Two PCR amplification reactions were set up for each sample. The component of the two reactions was identical except for the respective allele-specific reverse primers, one matching to G and the other to A. The real-time PCR was carried out on a Step one plus (ABI) by a 25 ul reactions mixture of 12.5 ul Power SYBR Green PCR Master Mix (ABI), 5 pmol of each primer and 1 ul genomic DNA. The amplification conditions were as follows: 50°C for 2 min, 95°C for 10 min, followed by 40 cycles of denaturation at 95°C for 15 sec, annealing/extending at 58°C for 1 min. In the wake of PCR amplification ongoing, the fluorescence intensity of SYBR Green increased. Because of mismatching to allele, the efficiency of PCR amplification was diversity, resulting in different cycle threshold (Ct) value. Allele discrimination of rs671 was successfully achieved based on the difference between the Ct values from the two reactions of each sample.

### 5 Pyrosequencing

Two primers for target DNA amplification and one primer for sequencing were designed by PyroMark Assay Design 2.0 (Qiagen). The 5′ base of the forward primer was labeled with a biotin (Table 1). PCR amplification reactions contained 5 μl 10x Buffer, 4 μl dNTP, 0.25 U Taq DNA Polymerase (TaKaRa Biotechnology, Tokyo, Japan), 1 ul genomic DNA, and 40 pmol of amplification primers in a reaction volume of 50 μl. PCR conditions were as follows: initial denaturation at 95°C for 5 min, followed by 40 cycles of 95°C for 30 sec, 62°C for 30 sec, 72°C for 10 sec, and finally extending 72°C for 7 min. Following PCR, the fragments were checked by 1% agarose gel electrophoresis. The single-stranded PCR product labeled with a biotin was purified and tested on a PyroMark Q24 (Qiagen) according to the manufacturer’s recommendations.

### 6 Sanger sequencing

To verify the genotype of rs671, Sanger sequencing was also carried out as our reference method using ABI 3130xl capillary sequencer (Applied Biosystems, Foster City, CA). The sequences of primers are shown in Table 1. The sequence was detected by Chromas program (Technelysium Pty. Ltd, Helensvale, Queensland, Australia).

## Results

### 1 Results of HRM

**Fig 1:**
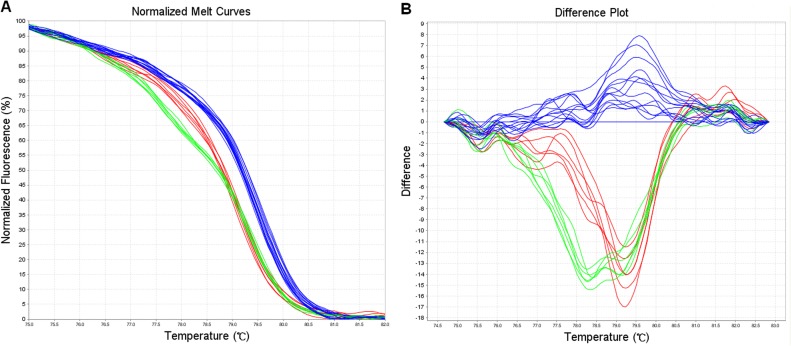
Typical results in genotyping ALDH2 by HRM. A. Normalized melt curves; B. Difference plot. Curves of different colors indicated samples of GG (blue), GA (red) and AA (green), respectively.

As shown in Fig 1, three genotypes of rs671 (GG, GA and AA) could be clearly distinguished using HRM. Different from previous reports [6], instead of sequence-specific hybridization probes, a saturating DNA dye SYTO 9, embedding DNA duplexes, was added to the PCR reaction mixture, which was much simpler and more convenient. Fluorescent signal gradually enhanced with the increase of the amplification product. Subsequently the signal receded following transition of DNA duplexes to single stranded DNA due to a ramp rate of 1% thermal denaturation. This method discriminates alleles at a SNP according to fluorescent signal changes during the course of thermal denaturation. If the difference between melting profiles of each allele was sufficient, amplification and genotyping can be simultaneously completed in one single tube without any post-PCR procedure. It has been reported that HRM was more applicable for class 1 (A>C, A>G) SNPs genotyping such as ALDH2 rs671 (G>A) [12]. It is worth noting that well designed primers, smaller amplicon, optimal melting protocol setting and data analysis are all critical for reliable discrimination of rs671 by HRM [13].

### 2 Results of TaqMan

**Fig 2:**
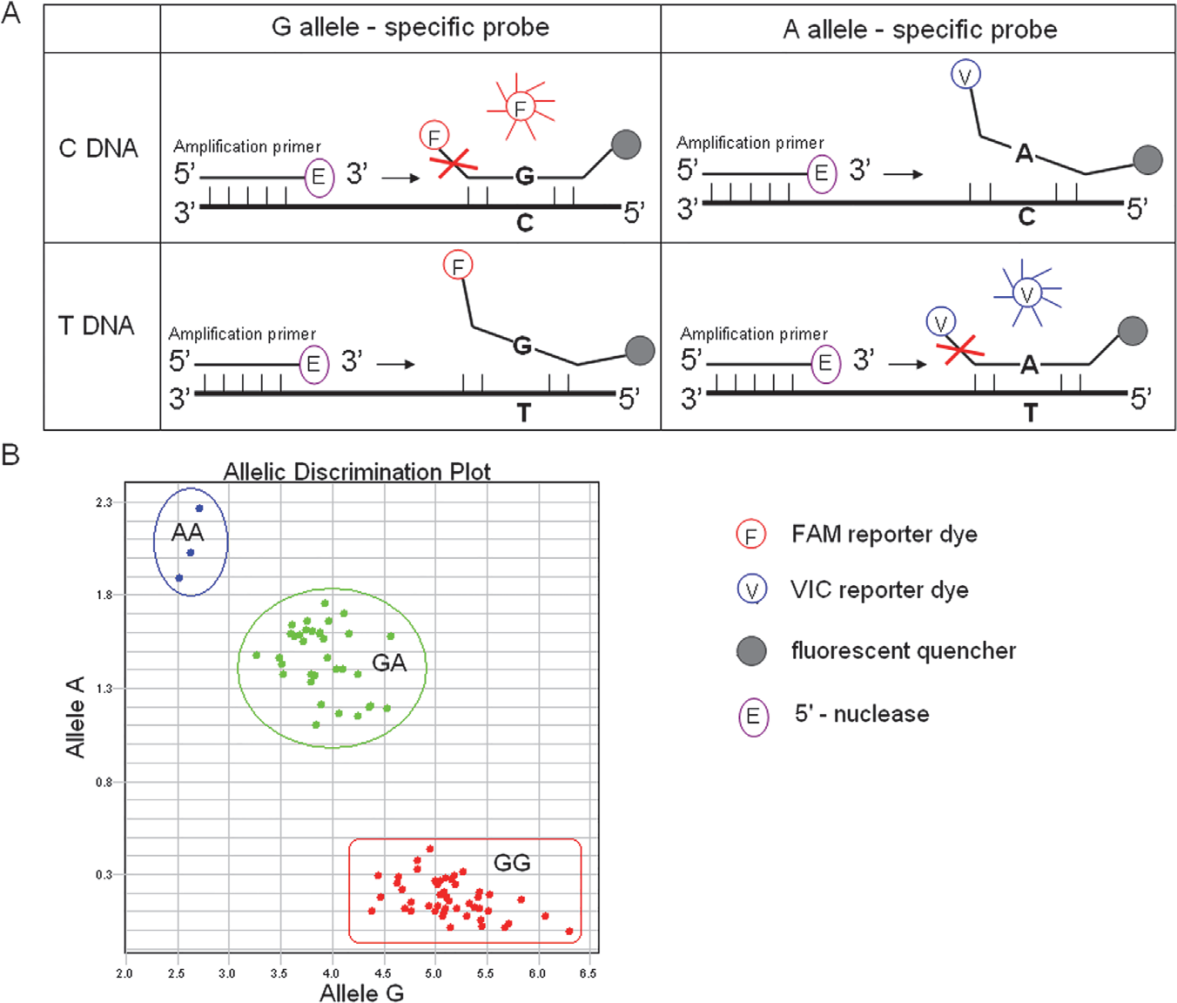
The principle and rs671 genotyping results of TaqMan. A. When the allele-specific probes were completely hybridized to template DNA, Taq polymerase (having the 5’—nuclease activity) cleaved the reporter dye and emit fluorescence. However, when a single basic mismatch existed between probes and template DNA, the hybridization was ineffective and the corresponding reporter dye couldn’t emit fluorescence adequately. B. Based on the fluorescence signal intensity of FAM and VIC (HEX replaced VIC in this research), samples were automatically classified into three groups corresponding to genotypes GG, GA and AA of rs671.

Fig 2 showed the principle and rs671 genotype results of TaqMan. Briefly, two allele-specific probes with only one single basic difference were labeled with a fluorescent reporter dye (FAM and VIC, respectively) as well as a quencher molecule. The quencher molecule suppresses the fluorescence signal of the reporter dye when the probes are intact. The allele-specific probes, which were completely hybridized to DNA templates during the annealing process, were cleaved by the 5’—nuclease activity of Taq polymerase, separating the reporter from quencher, and permitted the reporter dye to emit fluorescence. However, the hybridization between probes and template DNA were destabilized as long as a single basic mismatch existed between them, and corresponding reporter dye couldn’t emit fluorescence adequately [10] (Fig 2A). On the basis of fluorescence signal intensity of FAM and VIC (HEX replaced VIC in this research), samples were automatically classified into three groups corresponding to genotypes GG, GA and AA of rs671 (Fig 2B). We applied DNA minor groove binders to improve the specificity of TaqMan assays.

### 3 Results of AS-PCR

**Fig 3:**
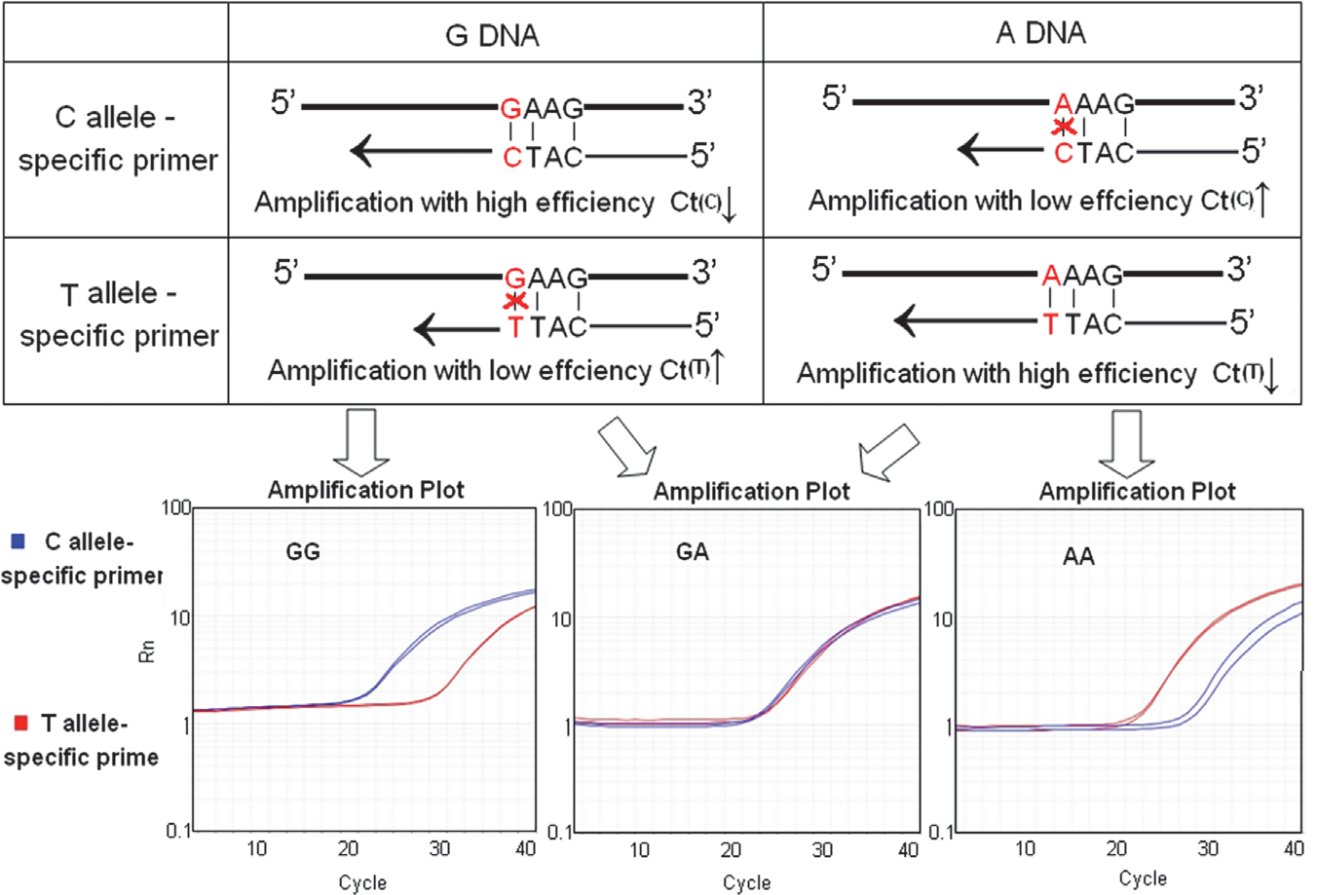
The principle and rs671 genotyping results of AS-PCR. A. The binding efficiency of allele-specific primer to target sequence was high when they matched well at the 3′ end of the primer, resulting in efficient amplification with smaller Ct (C-G; T-A). Vice versa, the efficient amplification is low due to the mismatch between the primer and the target sequence, resulting in larger Ct. B. Samples could be distinctly detected upon different Ct value of the two tubes. A genotyping result of GG was shown that the Ct value of the tube containing C allele-specific primer was smaller than the other one comtaining G allele-specific primer.

We also tested the performance of AS-PCR for genotyping ALDH2 (Fig 3). Different from above-mentioned methods, each sample was detected in at least two vials with different reverse allele-specific primers by AS-PCR. PCR reactions of the two vials had no difference except for the allele-specific primer, one matching to allele G and the other to A of rs671. The binding efficiency of allele-specific primer to target sequence was high when they matched well at the 3′ end of the primer, resulting in efficient amplification with smaller Ct. In contrast, the efficiency of amplification was low with mismatch between the primer and the target sequence, resulting in larger Ct (Fig 3A). Samples could be distinctly detected upon different Ct value of the two vials (Fig 3B). To increase the allele-specificity, we introduced an additional primer mismatch at the third nucleotide from the 3' end of the primers.

### 4 Results of pyrosequencing

**Fig 4:**
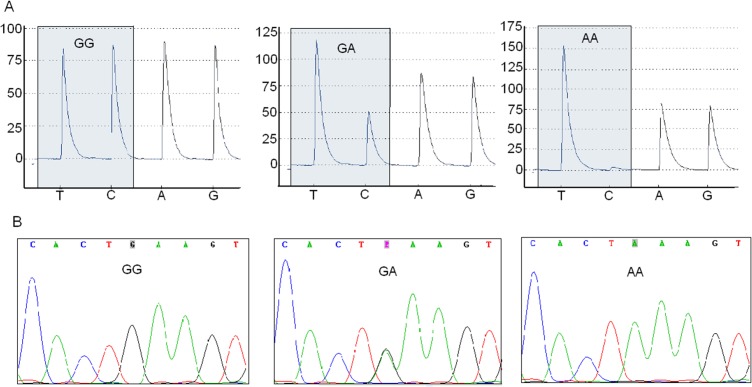
Typical results in genotyping ALDH2 by pyrosequencing and Sanger sequencing. A. Three genotypes of rs671 presented different peaks by pyrosequencing. B. Genotyping results were verified by Sanger sequencing.

In this study, we also chose pyrosequencing to genotype ALDH2. This technique is based on the detection of ATP generated by pyrophosphate. After PCR, single DNA strand labeled with a biotin was purified, separated, and used as sequence template. The dNTPs loaded in four holes were added to reaction one by one and pyrophosphate was released on condition that the incoming dNTP was complementary to the template. As a result, on the presence of enzyme ATP Sulfurylase and Luciferase, the reaction produced ATP and generated detectable lights shown as a peak on the pyrogram (Fig 4A). Finally, we used Sanger sequencing (current gold-standard) to verify the results obtained from the above-mentioned methods (Fig 4B).

## Discussion

Detection technology used for clinical trials or routine testing in diagnostic laboratories must meet the following key requirements: accurate—results should be concordant with an established laboratory method; affordable—inexpensive for patients; user-friendly—minimal hands-on operation; rapid—short turnaround time; simple—no complex equipment. In this study, ALDH2 genotypes were successfully determined in all 96 samples by referred four different methods (HRM, TaqMan, AS-PCR and pyrosequencing), and results were concordant with the results of Sanger sequencing.

One significant advantage of HRM over other methods is that HRM is a simple as well as cost effective method for genotyping ALDH2. The operation can be completed in one single tube and there is no need of costly probe synthesis and labeling. It is also time-saving (completed in about two hours) and has low risk for DNA contamination. Therefore, it is highly applicable for testing clinical samples. Although the slight diversity in certain SNP allele types (A/T or C/G) may complicate the melting curves interpretation which limits HRM application, it is applicable for class 1 SNPs genotyping such as ALDH2 rs671 (G>A) [12]. And our results demonstrated that HRM is a reliable method for genotyping ALDH2 rs671. Besides genotyping, HRM was also used for gene scanning and methylation analysis [14, 15]. One current limitation of HRM is the possibility that another unexpected SNP interferes in the target SNP. Hence, we have designed amplicons that are as short as possible in order to limit the probability of such an event. In addition, an experienced and skilled technical staff for final analysis can improve the efficiency of the test.

TaqMan is a rapid and very effective tool for genotyping especially for high throughput genetic studies. Similar to HRM, the PCR and allele detection are integrated into one process by TaqMan minimizing manual or robotic handling steps. The ability to automate data handling further enhances accuracy by eliminating operator bias and reduces labor intensity. In addition to detecting SNPs, small gene deletions and insertions can be identified by this method [16]. However, the cost of allele-specific probes is considerably high (about 4000 RMB for one SNP). What’s worse, the probes become quite unstable after repeated freezing/ thawing, which is not suitable for determining multiple SNPs in a small number of samples.

AS-PCR significantly reduces the cost by using SYBR Green which is much cheaper than SYTO 9 used in HRM. Without nonspecific fluorescence, this method can be used to genotype a large panel of informative biallelic SNPs. However, AS-PCR must be performed in separate wells and requires two reactions for each assay, doubling the amount of reagent and hands-on operation. To overcome this limitation, AS-PCR can be combined with HRM in practice with two key components enhancing PCR genotyping in the same tube [17]. Another limitation is that AS-PCR depends on the nature of SNPs and the surrounding sequences, with guanine cytosine-rich regions making PCR assays difficult to optimize [18].

Pyrosequencing involves three major steps: (1) PCR amplification of the region with target SNPs; (2) purification of PCR products; (3) sequence determination, thus increasing the time required and manual operation. Pyrosequencing requires specialized equipment and reagents. In addition, the requirement of four enzymes (DNA Polymerase, ATP Sulfurylase, Luciferase and Apyrase) increases its cost and enzymes are damageable, which limits its clinical application. Pyrosequencing not only distinguishes biallelic SNPs but also offers precise allele sizing which allowed it to be adopted in oncology.

DNA sequencing is the most direct diagnostic method to determine a target gene and remains the gold standard for genotyping, while it’s expensive and complex. In addition, an experienced and skilled technical staff is needed for the test.

Overall, HRM and AS-PCR are relatively straightforward and useful when dealing with a small number of samples usually a few hundred. The instrumentation used in HRM, TaqMan and AS-PCR is common and readily available, supported by Applied Biosystems (Foster City, CA, USA). All the above mentioned approaches rely on a PCR amplified genomic target as a substrate for the genotyping reaction and further technical improvements are needed to overcome this bottleneck.

## Conclusions

In conclusion, all four studied techniques are reliable methods for genotyping ALDH2 rs671. For routinely testing small number of clinical samples, HRM and AS-PCR outperform other two methods namely TaqMan and pyrosequencing. Relatively, TaqMan is optimal for high throughput genetic studies while pyrosequencing or Sanger sequencing are best reserved for verifying the test results when necessary.
